# An N-terminal transport-deficient module of CATION EXCHANGER1 is sufficient to trigger anoxia stress responses

**DOI:** 10.1093/plphys/kiaf353

**Published:** 2025-08-20

**Authors:** Hormat Shadgou Rhein, Shayan Sarkar, Antonella Gradogna, Gabrielle Durocher, Joachim Scholz-Starke, Jon K Pittman, Kendal D Hirschi

**Affiliations:** Pediatrics-Nutrition, Children's Nutrition Research, Baylor College of Medicine, Houston, TX 77030, USA; Pediatrics-Nutrition, Children's Nutrition Research, Baylor College of Medicine, Houston, TX 77030, USA; Department of Biological Sciences, University of Texas at El Paso, El Paso, TX 79968, USA; Institute of Biophysics, CNR—National Research Council of Italy, Genova 16149, Italy; Pediatrics-Nutrition, Children's Nutrition Research, Baylor College of Medicine, Houston, TX 77030, USA; Institute of Biophysics, CNR—National Research Council of Italy, Genova 16149, Italy; School of Natural Sciences, Faculty of Science and Engineering, The University of Manchester, Manchester M13 9PT, UK; Pediatrics-Nutrition, Children's Nutrition Research, Baylor College of Medicine, Houston, TX 77030, USA; Department of Biological Sciences, University of Texas at El Paso, El Paso, TX 79968, USA

## Abstract

Expressing a transport-deficient portion of a calcium transporter protein restores stress sensitivity in plants, revealing a signaling role independent of calcium movement.

Dear Editor,

Vacuolar cation exchangers (CAXs) act as calcium (Ca²⁺) transporters regulating cellular Ca²⁺ homeostasis and playing crucial roles in plant growth and stress responses (Pittman and Hirschi 2016, 2024). All CAX isoforms, including *Arabidopsis thaliana* (thale cress) CAX1, consist of 2 pseudosymmetrical modules connected by a hydrophilic loop and preceded by an N-terminal autoinhibitory domain (Pittman and Hirschi 2001). Genetic engineering to functionally modify these transporters can positively alter plant development, stress adaptation, and the nutritional quality of crops (Shigaki and Hirschi 2006; Hirschi 2008). When CAX1 is split into the 2 half proteins ½N-sCAX1 [the short N terminal 236 amino acid (AA) module of CAX1 lacking the autoinhibitory domain and comprising transmembrane helices (TM) 1 to 6] and ½C-CAX1 (the C-terminal 220 AA module of CAX1 comprising TM 7 to 11) and co-expressed in yeast, the modules can reconstitute a functional transporter, but the individual modules are nonfunctional in yeast due to lack of transport ability (Zhao et al. 2009). However, whether distinct structural modules of CAX1 can mediate signaling independent of Ca²⁺ transport has not been tested *in planta*.

Prior work demonstrated that an N-terminal CAX1 module containing the autoinhibitory domain can act as a dominant-negative regulator of native CAX function, when expressed in wild-type *Arabidopsis* (Sarkar et al. 2024). Loss-of-function *Arabidopsis cax1* mutants are more tolerant to anoxia (oxygen depletion), indicating that changes to cytosolic Ca^2+^ signaling may be involved in this response (Yang et al. 2022; Mathew et al. 2024), but whether CAX1-mediated Ca^2+^ transport is critical to anoxia responses is unclear. This anoxia phenotype can now be used to express CAX variants, including split CAX modules, in the *cax1* knockout background, to identify factors that phenocopy CAX1 activity.

To explore the signaling potential of CAX1 independent of transport, we expressed the synthetic construct ½N-sCAX1 (Fig. 1A) in the *cax1* background under the control of the strong *CaMV-35S* promoter or the native *CAX1* promoter (Supplementary Table S1). Most independent lines (∼85%) expressing *35S::½N-sCAX1* in *cax1*, including *35S::½N-sCAX1*#8 and *35S::½N-sCAX1*#13, restored sensitivity to anoxia (Supplementary Fig. S1, Fig. 1B). Phenotypes included post-hypoxic chlorophyll loss (Fig. 1C), increased hydrogen peroxide (H_2_O_2_) accumulation (Fig. 1D), and elevated malondialdehyde levels (Fig. 1E) in comparison to *cax1* mutant, phenocopying wild-type (Col-0) responses. In contrast, *½N-sCAX1* expressed under the native *CAX1* promoter failed to produce these phenotypes (Fig. 1F). Reverse transcription quantitative PCR analyses showed that the *½N-sCAX1* module under the *35S* promoter exhibited approximately 10 to 15 times higher expression levels than when driven by the *CAX1* promoter, suggesting that elevated expression of ½N-sCAX1 is required for promoting anoxia sensitivity (Fig. 1G). Plants expressing *35S::½N-sCAX1* in the *cax1* background also displayed elevated reactive oxygen species detected by diaminobenzidine staining, increased electrolyte leakage and water loss, indicating membrane damage following reoxygenation (Supplementary Fig. S2). These physiological indicators were intermediate between Col-0 and *cax1*, indicating a partial restoration of post-anoxia stress signaling.

**Figure 1.**
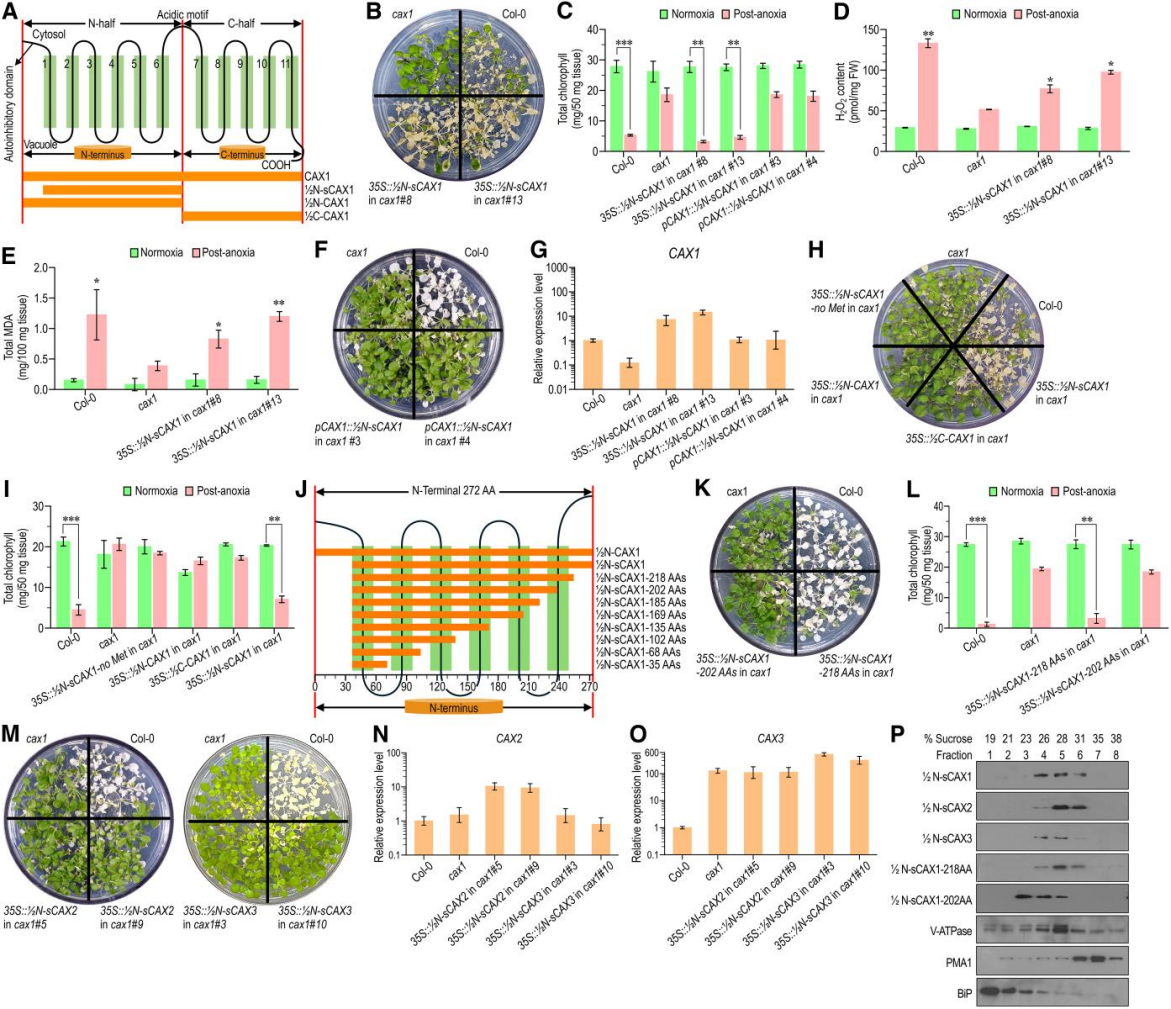
Short N-terminal module of CAX1 without autoinhibitory domain restores anoxia sensitivity in *cax1* mutants. **A)** Schematic representation of Arabidopsis CAX1 transporter showing TMs (1 to 11), the autoinhibitory domain, and the acidic motif loop separating the N- and C- terminal halves and the coverage of full-length CAX1, ½N-CAX1, ½N-sCAX1, and ½C-CAX1 indicated as horizontal bars. **B)** Anoxia suppression phenotype displayed by 2 independent lines of *cax1* mutant expressing *35S::½N-sCAX1*. Plates were grown under normal growth conditions for 21 d and were placed under anoxic conditions for 8 h. Plants were returned to normal growth conditions; pictures were taken 3 d after anoxia treatment. **C)** Chlorophyll content in normoxia and post-anoxia conditions in the transgenic lines expressed in *cax1* compared to Col-0 and *cax1*. **D)** Internal H_2_O_2_ (expressed as pmol H_2_O_2_/mg fresh weight) production assays were performed using Col-0, *cax1* mutant, *cax1* expressing *35S::½N-sCAX*, and the fluorescence intensity was measured by excitation at 530 nm and emission at 590 nm. **E)** Malondialdehyde contents in Col-0, *cax1* mutant, *cax1* expressing *35S::½N-sCAX* plants before anoxia (normoxia) and post-anoxia. **F)** Anoxia suppression phenotype displayed by 2 lines of *cax1* mutant expressing *pCAX1::½N-sCAX1.* The assay was performed as described in (B). **G)** Relative quantification of *CAX1* expression in transgenic *cax1* lines in comparison to Col-0 and *cax1*. *UBQ10* was used as an internal control. *CAX1* expression in Col-0 was set to 1. **H)** Anoxia assay of *cax1* lines expressing *35S::½N-sCAX1-no Met*, *35::½N-CAX1*, and *35S::½C-CAX1* and performed as described in (B, C). **I)** Chlorophyll content in normoxia and post-anoxia conditions in *cax1* plants expressing the constructs displayed in **(H)**. **J)** Schematic of deletion constructs of ½N-sCAX1 from the C-terminal end, keeping the N-terminal end constant in each construct. **K)** Anoxia assay showing *cax1* line expressing *35S::½N-sCAX1-218 AAs* is anoxia sensitive, while *cax1* expressing *35S::½N-sCAX1-202 AAs* is anoxia-tolerant. **L)** Chlorophyll content in normoxia and post-anoxia conditions in *cax1* plants expressing *½N-sCAX1-218 AAs* and *½N-sCAX1-202 AAs* compared to *cax1* and Col-0. **M)** Anoxia assay displaying *cax1* lines expressing *35S::½N-sCAX2* (left) and *35S::½N-sCAX3* (right) were anoxia tolerant. **N)** and **O)** Relative quantification of *CAX2* transcript levels (*M*) and *CAX3* transcript levels (*N*) in Col-0, *cax1* and 2 lines of *cax1* expressing *35S::½N-sCAX2* and *35S::½N-sCAX3. UBQ10* was used as an internal control. The expression levels of the indicated genes in Col-0 were set to 1. For all graphs, data are mean values ± SD from 3 biological and 4 technical replicates. Asterisks indicate significant difference from the pretreatment conditions (***P < 0.001, **P < 0.01, and *P < 0.05; according to 2-way ANOVA followed by Bonferroni's post hoc test). **P)** Western blots showing subcellular localization of C-terminal myc-tagged half N-terminal CAX modules (½N-CAX1, ½N-sCAX1 ½N-sCAX2, ½N-sCAX3, ½N-sCAX1-218 AA, and ½N-sCAX1-202 AA) in *N. benthamiana*. The fractions [fraction 1 = 19%; fraction 8 = 38% (v/v) Suc] were subjected to Western blot analyses using antibodies against myc (half CAX modules) and plant membrane markers: the plant ER luminal protein (BiP; Joseph et al. 2012), vacuolar ATPase (V-ATPase; Ward et al. 1992), and a PM H^+^-ATPase (PMA1; Villalba et al. 1992) according to Hirschi et al. (2000).

Overexpression of a no-start-codon version of ½N-sCAX1 (*35S::½N-sCAX1-no Met*) in *cax1* had no effect, confirming that the anoxia phenotype depends on protein production rather than transcript accumulation (Fig. 1H). Moreover, constructs containing the N-terminal half of CAX1 but with the autoinhibitory domain (½N-CAX1; Fig. 1A) or the C-terminal half of CAX1 (½C-CAX1), despite high expression (Supplementary Fig. S3), failed to confer anoxia sensitivity in *cax1* (Fig. 1H) as confirmed by similar chlorophyll levels with *cax1* (Fig. 1I). To determine structural determinants of this effect, we generated truncations of ½N-sCAX1 (Fig. 1J). Constructs containing all 6 predicted TM domains retained function (½N-sCAX1-218 AA), while those lacking one or more TM helices failed to restore anoxia sensitivity (Fig. 1K, Supplementary Fig. S4) as evidenced by chlorophyll measurements (Fig. 1L). This was despite all constructs having similar expression (Supplementary Fig. S5) and tonoplast localization (Fig. 1P, Supplementary Fig. S6). These results suggest that the process that initiates anoxia sensitivity requires just the N-terminal 6-helix core of CAX1, that the autoinhibitory domain suppresses this process, and that the acidic motif (TM6 to TM7 linker) was dispensable. Homologous N-terminal module variants of CAX2 and CAX3 did not restore sensitivity (Fig. 1M, left and right), despite high expression levels (Fig. 1, N and O) and correct tonoplast targeting (Fig. 1P, Supplementary Fig. S6), highlighting the functional specificity of the CAX1 N-terminal module.

We used combined patch-clamp and fura-2 fluorescence recordings in isolated vacuoles from *35S::½N-sCAX1*/*cax1* lines (Gradogna et al. 2009; Mathew et al. 2024) to assess Ca²⁺ transport activity. Overexpression of ½N-sCAX1 did not restore vacuolar Ca²⁺ transport (Fig. 2, A and B), reinforcing that the construct is transport-deficient. This conclusion was further supported by suppression of Ca^2+^ sensitivity of the yeast (*Saccharomyces cerevisiae*) strain K667 (*cnb1::LEU2 pmc1::TRP1 vcx1*Δ), deficient in vacuolar Ca^2+^ transport (Cunningham and Fink 1996) showing ½N-sCAX1 is non-functional in yeast complementation assays (Zhao et al. 2009; Supplementary Fig. S7), strongly demonstrating that the ½N-sCAX1 module by itself is not capable of high-capacity Ca^2+^ transport.

**Figure 2.**
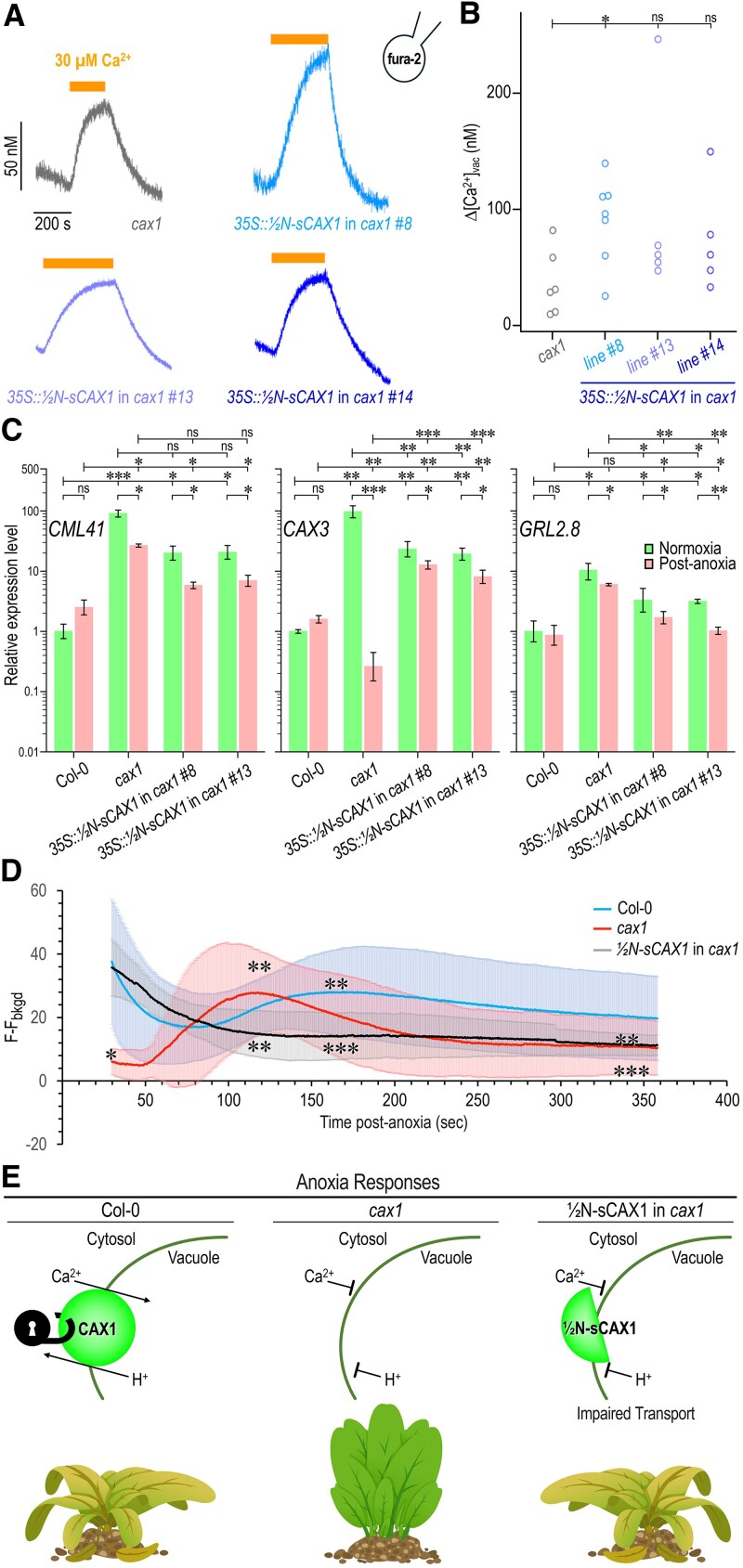
½N-sCAX1 alters Ca²⁺-responsive signaling without demonstrated transport. **A)** Representative fura-2 fluorescence ratio recordings performed on isolated vacuoles from *cax-1* plants and 3 independent *35S::½N-sCAX1* lines with ascertained high transgene expression, in response to bath application of 30 *µ*M Ca^2+^ (indicated by *orange bars*). Vacuoles were pre-loaded with 100 *µ*M fura-2 through the patch pipette and kept at a membrane voltage of −40 mV. Vacuolar fluorescence ratio values were converted into [Ca^2+^] units. Col-0 was excluded from the data due to ectopic *CAX3* upregulation in *cax1* mutant, and because of the different promoters (endogenous promoter vs *35S*) used. **B)** Summary plot of the amplitudes of vacuolar [Ca^2+^] changes (*Δ*[Ca^2+^]*_vac_*) evoked by bath application of 30 *µ*M Ca^2+^. Data points represent measurements on individual vacuoles derived from 3 to 4 different plants per line. Color code as in (A). Statistical significance: *cax1* vs line #8: *P = 0.0135 (Student's *t*-test); *cax1* vs line #13: ns (not significant) *P* = 0.1021 (Wilcoxon Rank test); *cax1* vs line #14: ns, *P* = 0.1614 (Student's *t*-test). **C)** Ca^2+^ dependent gene expression (*CML41*, *CAX3*, and *GLR2.8*) in transgenic lines: *35S::½N-sCAX1* in *cax1*#8 and *35S::½N-sCAX1* in *cax1*#13, wild type Col-0, and *cax1* under normoxia and post-anoxia condition. *UBQ10* was used as an internal control. The expression levels of the indicated genes in Col-0 at normoxia were set to 1. Data are mean values ± SE of 3 biological replicates. Asterisks indicate significant expression difference from Col-0 and *cax1* (***P < 0.001, **P < 0.01, *P < 0.05, and ns, non-significant; according to 2-way ANOVA followed by Bonferroni's post hoc test). **D)** Quantification of fluorescence of the plants during reoxygenation after anoxia stress. Col-0, *cax1* and *35S::½N-sCAX1* in *cax1* stably expressing the genetically encoded Ca^2+^ biosensor (GCamP3) were grown on ½ MS for 14 d under normal growth conditions. Plants were treated with anoxia stress for 4 h. The intensity of fluorescence of GCamP3 was recorded immediately after reoxygenation using a fluorescence microscope. Data are representative of ≥3 independent plants/leaves. The error bars are means ± SD from 3 biological replicates. Asterisks indicate significant differences in Ca^2+^ signaling between Col-0, *cax1*, and *35S::½N-sCAX1* at 30, 120, 166, and 358 s (***P < 0.001, **P < 0.01, and *P < 0.05; according to paired *t*-test). **E)** Model showing divergent roles of CAX1 and an N-terminal truncated variant in calcium transport and shared mechanisms in anoxia responses. CAX1 is functional (in the wild type Col-0 background) when the autoinhibitory domain (represented by a lock) is “unlocked” by a trans-acting protein (Left). When the transporter is not functioning (such as when absent in the *cax1* knockout background), plants are anoxia-tolerant (Middle). CAX1 comprises 2 pseudosymmetrical modules indicated by semi-circles. When the ½N-sCAX1 module is expressed at high levels in *cax1*, it mimics an activated CAX1 phenotype to produce anoxia sensitivity by unknown mechanisms that appear to be independent of Ca^2+^ transport (Right).

To determine whether ½N-sCAX1 also affects Ca^2+^-responsive transcription, we measured expression of 3 Ca²⁺-regulated genes—*CML41* (Xu et al. 2017), *CAX3* (Manohar et al. 2011), and *GLR2.8* (Kim et al. 2001)—under normoxic and post-anoxic conditions (Supplementary Table S2). These transcripts are elevated in *cax1* during normoxia and suppressed in post-anoxia conditions (Yang et al. 2022). In our experiments, *cax1* again showed heightened baseline expression and a post-anoxia drop. The *35S::½N-sCAX1* lines exhibited intermediate expression patterns between *cax1* and Col-0, particularly post-anoxia, suggesting partial restoration of Ca²⁺-regulated gene expression (Fig. 2C). Furthermore, to assess whether the observed rescue of anoxia sensitivity (Fig. 1) was due to altered Ca²⁺ responsive signaling, we used a GFP-based Ca^2+^ biosensor (GCaMP3) to monitor cytosolic Ca²⁺ transients following anoxia, using the procedure described by Yang et al. (2022). In response to anoxia, *35S::½N-sCAX1* lines displayed partial restoration of the early Ca^2+^ spike typically seen in Col-0 (Fig. 2D, Supplementary Fig. S8). These results indicate that ½N-sCAX1 modifies Ca^2+^ signaling output in a manner that is uncoupled from CAX1-mediated Ca²⁺ transport.

Collectively, our findings indicate that overexpression of a truncated, transport-deficient form of CAX1 restores anoxia sensitivity and associated signaling outputs in *Arabidopsis* (Fig. 2E). The ½N-sCAX1 module restores phenotypes typically attributed to full-length CAX1 in the absence of measurable Ca²⁺ transport, suggesting that the TM1–TM6 region, when decoupled from the autoinhibitory domain, acts as a stress-responsive signaling module. Furthermore, the tonoplast localization of ½N-sCAX1 supports a model in which the N-terminal half of CAX1 functions as a signaling module within the cytosolic-vacuole region of the cell, possibly by scaffolding or modulating membrane complexes in response to stress. The fact that homologous CAX fragments do not produce similar effects reinforces the specificity of the CAX1 N-module in this non-transport role.

These findings extend prior studies of CAX1 dominant-negative modules (Sarkar et al. 2024) and suggest that removing the autoinhibitory domain exposes a latent, transport-independent signaling capacity for this N-terminal half of CAX1. This work highlights how membrane transporters may harbor multifunctional domains that uncouple ion flux from stress signaling—an insight with potential implications for synthetic biology and stress-resilient crop design.

## Supplementary Material

kiaf353_Supplementary_Data

## Data Availability

All materials and data are available upon request from the corresponding author.

## Supplementary data

The following materials are available in the online version of this article.

**Supplementary Figure S1.** Anoxia response of different independent T_2_ lines of *35S::½N-sCAX1* in *cax1*.

**Supplementary Figure S2.** Post-anoxia phenotypes resulting from elevated expression of *½N-sCAX1* in *cax1*.

**Supplementary Figure S3.** Relative quantification of *CAX1* transcript levels in Col-0, *cax1* and 2 lines of *cax1 35S::½N-sCAX1-no Met*, *35::½N-CAX1*, *35S::½C-CAX1*, and *35S::½N-sCAX1.*

**Supplementary Figure S4.** Anoxia response of *½N-sCAX1* 3′ deletions in *cax1* background.

**Supplementary Figure S5.** Relative quantification of *CAX1* transcript levels in Col-0, *cax1* and 3 lines of *cax1* mutant expressing 35S*::½N-sCAX1-218 AAs* and 35S*::½N-sCAX1-202 AAs*.

**Supplementary Figure S6.** Protein abundance of half N-terminal CAX modules *in planta*.

**Supplementary Figure S7.** Yeast suppression assay of ½N-sCAX1.

**Supplementary Figure S8**. Representative images of Ca signaling in plants expressing the GCaMP3 Ca^2+^ biosensor after 4 h anoxia at different time points (40 , 80 , 120 , 160 , 200, 240, and 280 s after reoxygenation).

**Supplementary Table S1.** List of constructs for generating transgenic lines in *cax1* alleles with forward and reverse primers and anoxia phenotypes.

**Supplementary Table S2.** List of reverse transcription-quantitative PCR primers used in the study.

## Dive Curated Terms

The following phenotypic, genotypic, and functional terms are of significance to the work described in this paper:

CAX1 Gramene: AT1G16380
CAX1 Araport: AT1G16380

CAX3 Gramene: AT3G51860
CAX3 Araport: AT3G51860

UBQ10 Gramene: AT4G05320
UBQ10 Araport: AT4G05320

CAX2 Gramene: AT3G13320
CAX2 Araport: AT3G13320
