# Supplementary material for: An N-terminal transport-deficient module of CATION EXCHANGER1 is sufficient to trigger anoxia stress responses
Source: Plant Physiol. 2025 Aug 20;198(4):kiaf353. doi: 10.1093/plphys/kiaf353 (PMC12366789; doi:10.1093/plphys/kiaf353)
Supplement: kiaf353_Supplementary_Data [file kiaf353_supplementary_data.pdf]

Supplementary Data

An N-terminal transport-deficient module of CATION EXCHANGER1 is sufficient to trigger anoxia stress responses

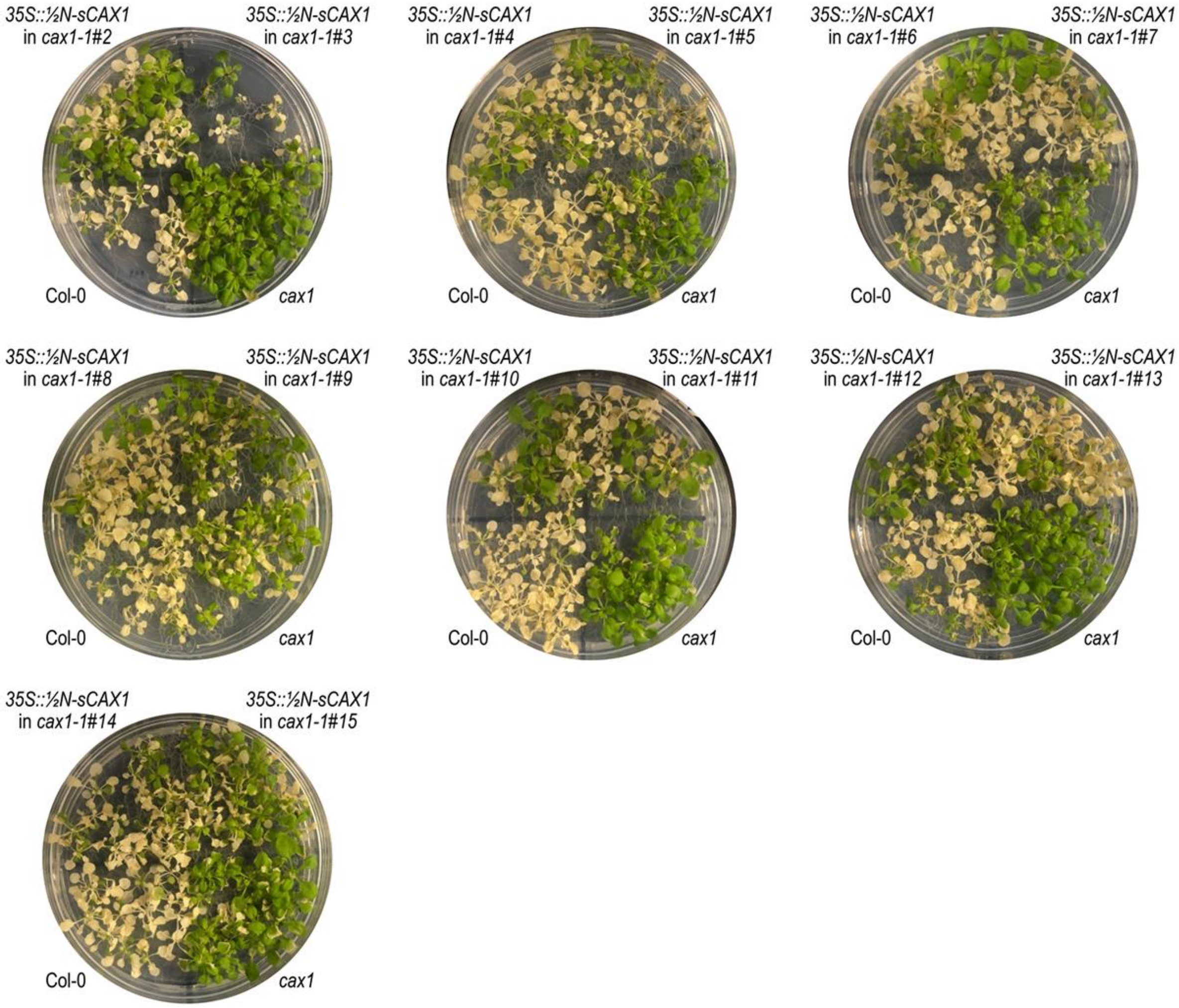

Supplementary Figure S1. Anoxia response of different independent T_2 lines of *35S*::*½N-sCAX1* in *cax1*. Around 13 lines of *35S*::*½N-sCAX1* showed mild to severe anoxia sensitivity. Plates were grown under normal growth conditions for 21 days and were placed under anoxic conditions for 8 hrs. Plants were returned to normal growth conditions, picture was taken 3 days after anoxia treatment.

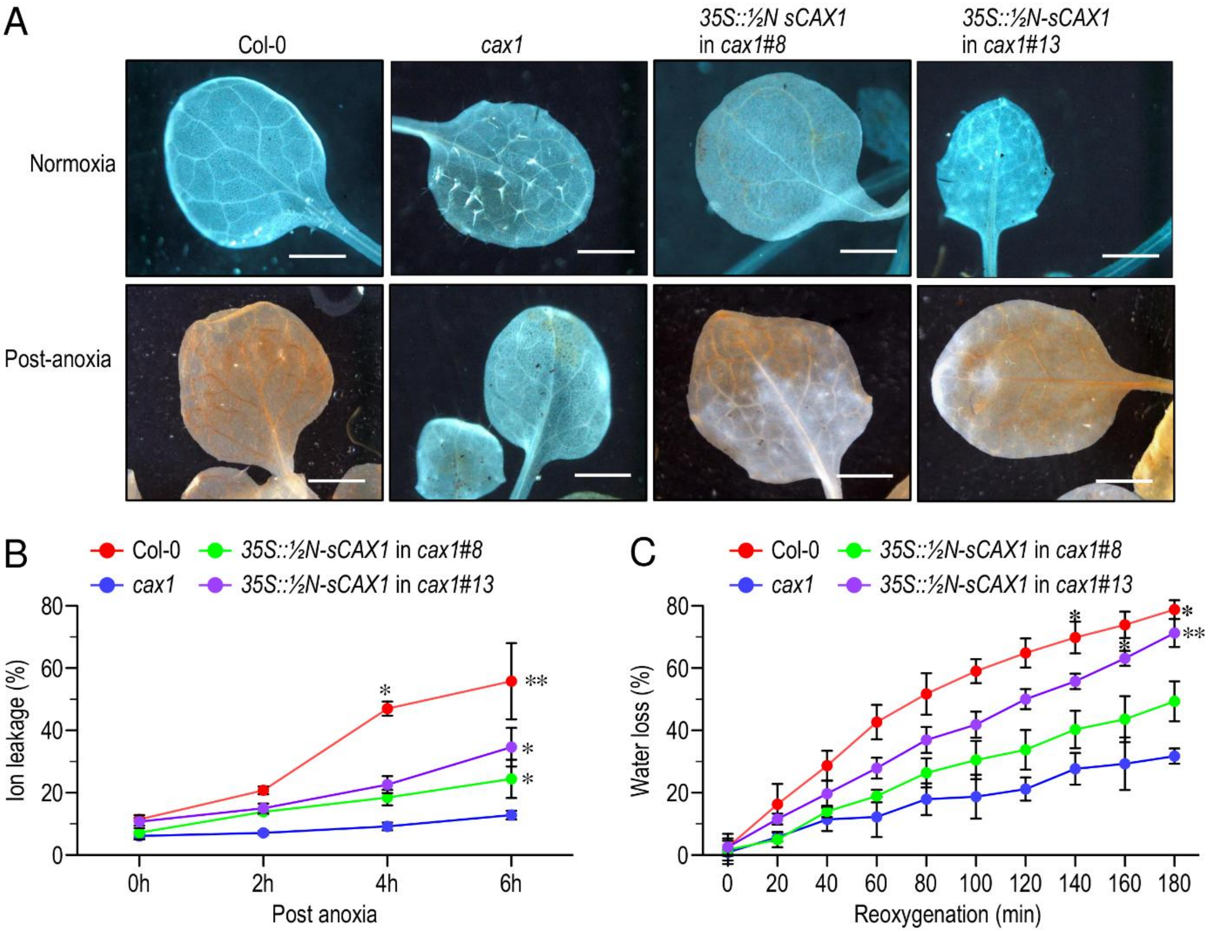

Supplementary Figure S2. Post-anoxia phenotypes resulting from elevated expression of ½N-sCAX1 in *cax1*. *A*, DAB staining showing levels of reactive oxygen species (ROS) in representative leaves of plants before anoxia (normoxia) and following post-anoxia. Strong ROS accumulation is determined by yellow-brown staining. Bars: 1 mm. *B*, Electrolyte leakage from Col-0, *cax1* and *35S*::*½N-sCAX1* in *cax1* plants after anoxia and reoxygenation for 2 h, 4 h and 6 h. Asterisks indicate significant differences from control (*, $P < 0.05$; **, $P < 0.01$; according to Student's *t*-test). Data are mean values ± SD from 3 biological replicates. *C,* Water loss of plants immediately after 8 h anoxia treatment (set to 0 min) and following reoxygenation over 180 min. Data are mean values ± SD from 3 biological replicates. Asterisks indicate significant differences from control (*, P < 0.05; **, P < 0.01; according to Student's t-test).

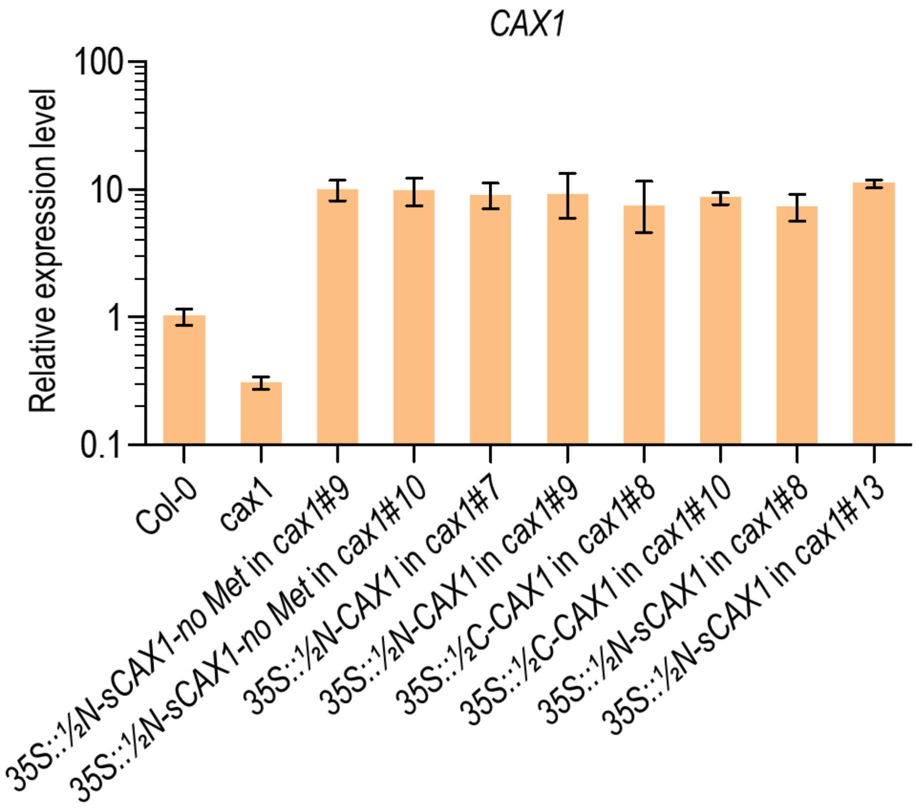

Supplementary Figure S3. Relative quantification of *CAX1* transcript levels in Col-0, *cax1* and 2 lines of *cax1 35S*::*½N-sCAX1-no Met*, *35*::*½N-CAX1*, *35S*::*½C-CAX1* and *35S*::*½N-sCAX1*. *UBQ10* was used as an internal control. *CAX1* expression in Col-0 was set to 1. Data are mean values ± SD of 3 biological replicates.

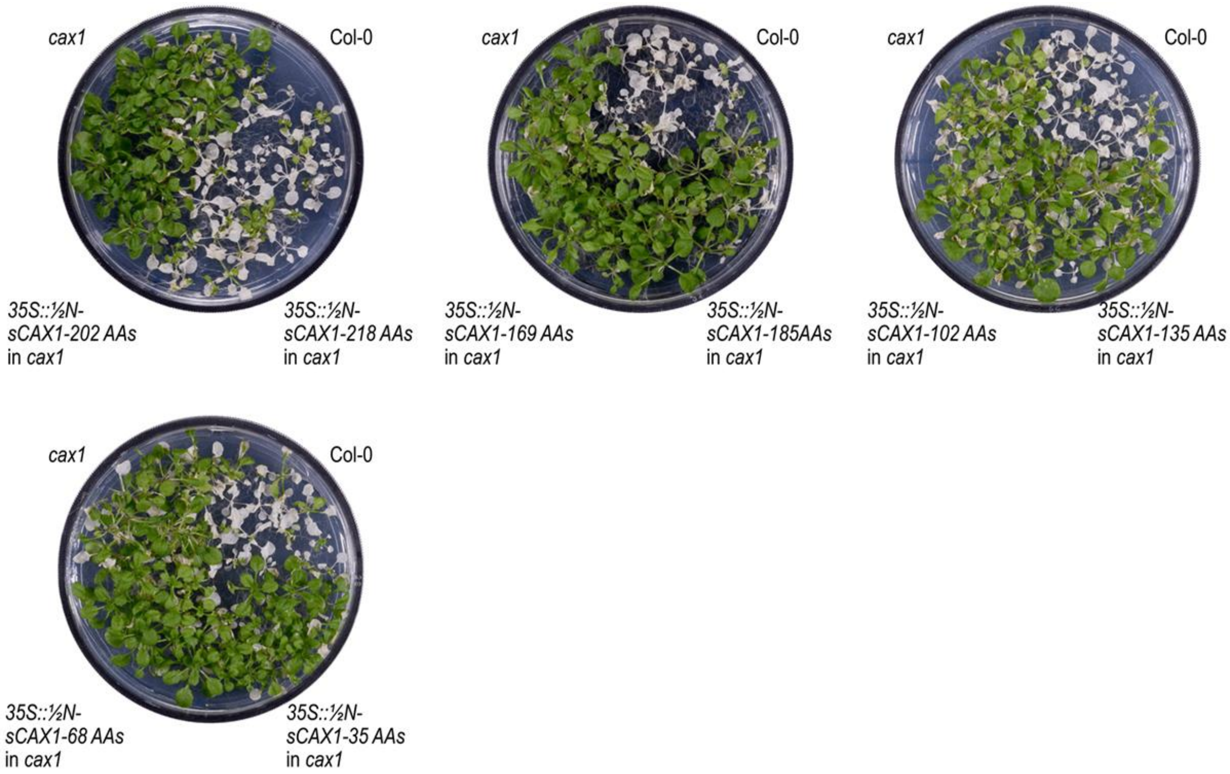

Supplementary Figure S4. Anoxia response of *½N-sCAX1* 3’ deletions in *cax1* background. The *cax1* mutant line expressing *35S::½N-sCAX1-218 AA* show anoxia sensitivity phenotypes while the *cax1* line expressing other deletions from *35S::½N-sCAX1-202 AA* to *35S::½N-sCAX1-35AA* were anoxia tolerant. Plates were grown under normal growth conditions for 21 days and were placed under anoxic conditions for 8 hrs. Plants were returned to normal growth conditions, picture was taken 3 days after anoxia treatment.

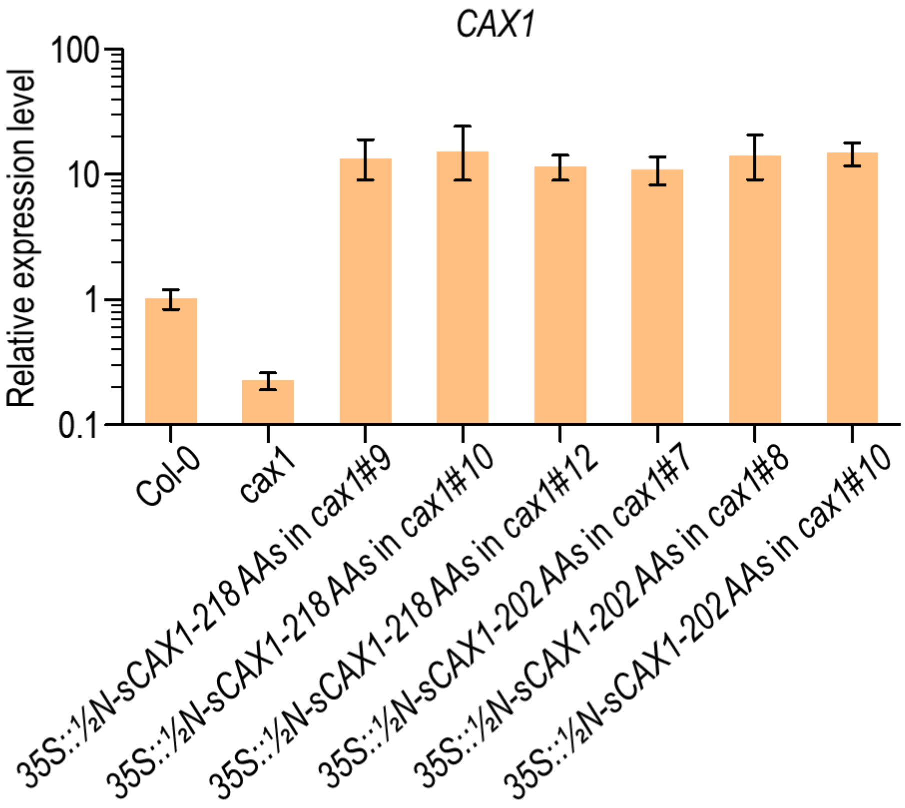

Supplementary Figure S5. Relative quantification of *CAX1* transcript levels in Col-0, *cax1* and 3 lines of *cax1* mutant expressing 35S::*½N-sCAX1- 218 AAs* and 35S::*½N-sCAX1- 202 AAs*. *UBQ10* was used as an internal control. *CAX1* expression in Col-0 was set to 1. Data are mean values ± SD of 3 biological replicates.

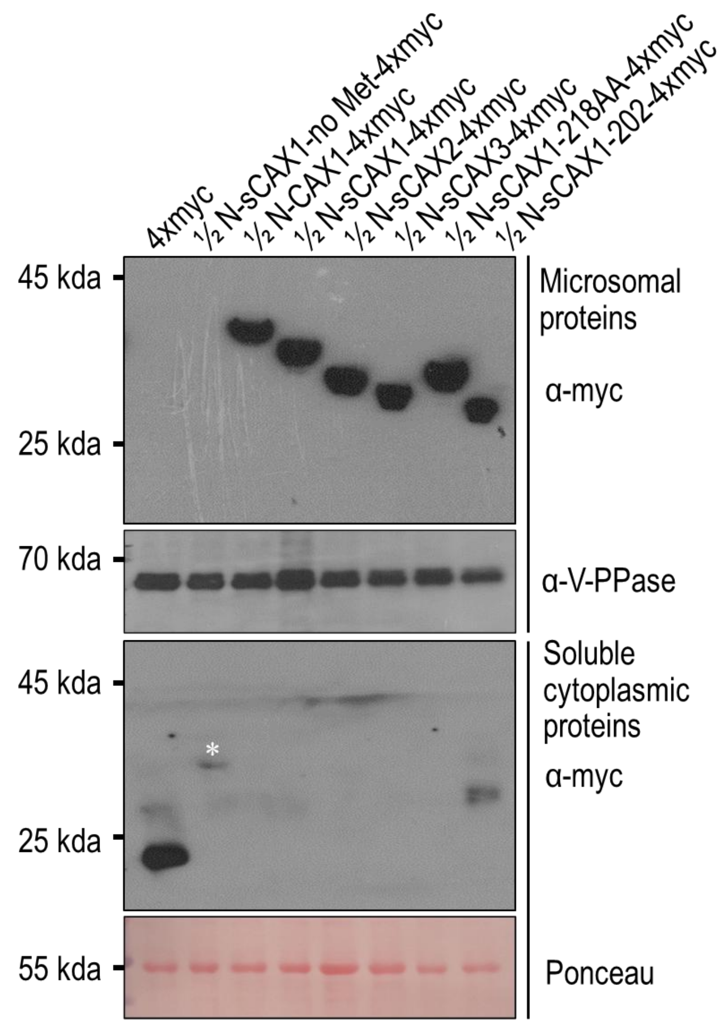

Supplementary Figure S6. Protein abundance of half N-terminal CAX modules *in planta*. Western blots showing relative levels of half N-terminal CAX modules (½N-CAX1, ½N-sCAX1, ½N-sCAX2, ½N-sCAX3, ½N-sCAX1-218 AA and ½N-sCAX1-202 AA) tagged with c-myc at the C terminus in microsomal fractions from *Nicotiana benthamiana* leaves, while low expression of ½N-sCAX1-202 AA was detected in the soluble fraction. ½N-sCAX1-no Met failed to produce any protein due to lack of a start codon. Microsomal and soluble fractions (25 µg) were run on 12.5% SDS-PAGE, blotted, and immunostained with antibody against c-*myc*. Equivalent protein loading and expression was determined by immunoblot analyses with the anti-V-PPase antibody (for microsomal proteins) and Ponceau S staining (for soluble proteins).

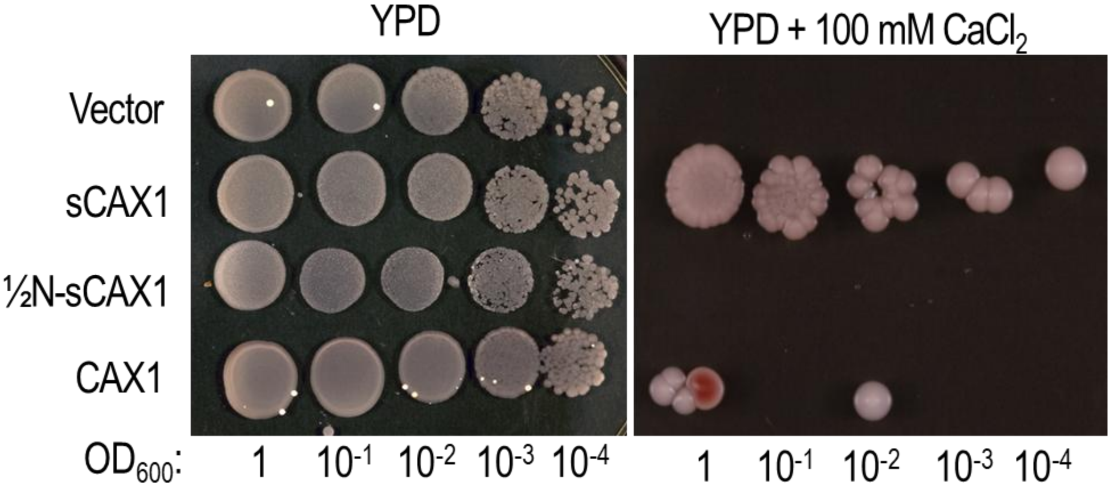

Supplementary Figure S7. Yeast suppression assay of ½N-sCAX1. Suppression of Ca^{2+} sensitivity of the *pmc1 cnb vcx1* yeast mutant (K667) by the various constructs, spotted onto yeast extract peptone dextrose (YPD) medium and the YPD medium containing 100 mM $CaCl_2$.

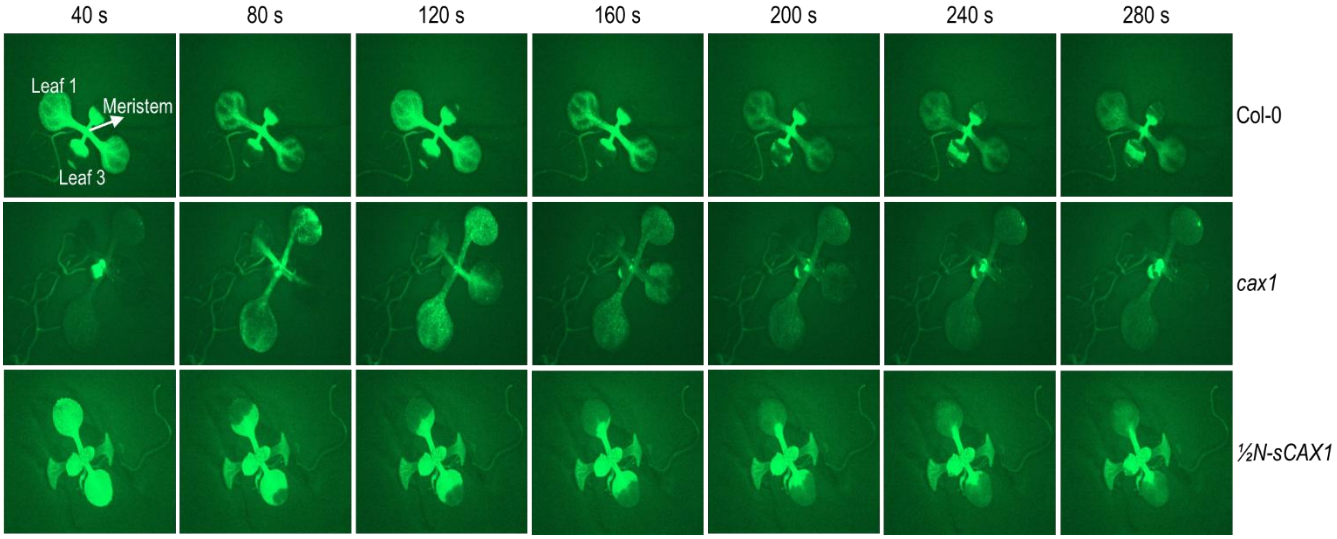

Supplementary Figure S8. Representative images of Ca signaling in plants expressing the GCaMP3 Ca^{2+} biosensor after 4 hr anoxia at different time points (40 s, 80 s, 120 s, 160 s, 200 s, 240 s and 280 s after reoxygenation). Col-0, *cax1* and *½N-sCAX1/cax1* stably expressing the genetically encoded Ca^{2+} biosensor, GCamP3 were grown on ½ MS for 14 days under normal growth conditions. Intensity of fluorescence of GCamP3 was recorded using a fluorescence microscope.

Supplementary Table S1. List of constructs for generating transgenic lines in *cax1* alleles with forward and reverse primers and anoxia phenotypes:

**Constructs**	**Forward Primer**	**Reverse Primer**	**Amino Acids**	**Background**	**Phenotype**
35S::½N-sCAX1	5`-ATGTCTTCTTCTTCTTTGAG-3`	5`-TTAATACTCATCTTCCTGTTCTTG -3`	236	*cax1*	Sensitive
pCAX1::½N-sCAX1	5`-ATGTCTTCTTCTTCTTTGAG-3`	5`-TTAATACTCATCTTCCTGTTCTTG -3`	236	*cax1*	Tolerant
35S::½N-sCAX-no Met	5`-TTATCTTCTTCTTCTTTGAG-3`	5`-TTAATACTCATCTTCCTGTTCTTG -3`	236	*cax1*	Tolerant
35S::½N-CAX1	5`-ATGGCGGGAATCGTGACAGAG-3`	5`-TTAATACTCATCTTCCTGTTCTTG -3`	272	*cax1*	Tolerant
35S::½C-CAX1	5`-ATGGATGACGATGTGGAGC-3`	5`-TTAAGATGAGAAAACTCCTCC-3`	191	*cax1*	Tolerant
35S::½N-sCAX1-218 AAs	5`-ATGTCTTCTTCTTCTTTGAG-3`	5`-TTAGAAAACAAGATATG-3`	218	*cax1*	Sensitive
35S::½N-sCAX1-202 AAs	5`-ATGTCTTCTTCTTCTTTGAG-3`	5`-TTATCGCGATATACTCAGTTGC -3`	202	*cax1*	Tolerant

35S::½N-sCAX1-185 AAs	5`-ATGTCTTCTTCTTCTTTGAG-3`	5`-TTAGTTTTTCAAGTATCCCAC -3`	185	*cax1*	Tolerant
35S::½N-sCAX1-169 AAs	5`-ATGTCTTCTTCTTCTTTGAG-3`	5`-TTATAGAAGTAAGAAGAAG-3`	169	*cax1*	Tolerant
35S::½N-sCAX1-135 AAs	5`-ATGTCTTCTTCTTCTTTGAG-3`	5`-TTACAATAAAAGGTTCGAC -3`	135	*cax1*	Tolerant
35S::½N-sCAX1-102 AAs	5`-ATGTCTTCTTCTTCTTTGAG-3`	5`-TTAGTTTCCACACGTTGCGTTC -3`	102	*cax1*	Tolerant
35S::½N-sCAX1-68 AAs	5`-ATGTCTTCTTCTTCTTTGAG-3`	5`-TTATAGCAAGCTAAGTCC -3`	68	*cax1*	Tolerant
35S::½N-sCAX1-35 AAs	5`-ATGTCTTCTTCTTCTTTGAG-3`	5`-TTAGAGAATGACTTCTTGG -3`	35	*cax1*	Tolerant
35S::½N-sCAX2	5`-ATGGAGCAAGGATCACTTTCTAC-3`	5`-TTATTCTTCGTTCTGATTTGATTCCTC -3`	231	*cax1*	Tolerant
35S::½N-sCAX3	5`-ATGTACAACTACGGTCGTCCGTTGA-3`	5`-TTAATCATATGCATCATCATCATCCTG-3`	185	*cax1*	Tolerant

Supplementary Table S2. List of RT-qPCR primers used in the study:

**Gene Name**	**ATG Identifier**	**Primer Sequence (5' → 3')**
UBQ10	AT4G05320	qF: GGCCTTGTATAATCCCTGATGAATAAG<br><br>qR: AAAGAGATAACAGGAACGGAAACATAGT
CAX1	AT2G38170	qF: GAAGATGAGTATGATGACGATGTGGAGCAAGAAA<br><br>qR: GTGGCTACAACATACTCCGATAG
CML41	AT3G50770	qF: TCCGTCAAGTCTTCAGCCAT<br><br>qR: TCAACTTCACCGTCACCGTA
CAX3	AT3G51860	qF: TGGGCCTGTTGTGTCATTTG<br><br>qR: AAACAATTGGCGGTGAGTCC
GLR2.8	AT2G29110	qF: TACATTCCGAGCCCTTCAAC<br><br>qR: CGTTCTTCATTCGGGGTAAA
